# A feasibility study of the internet-based intervention “Strategies for Empowering activities in Everyday life” (SEE 1.0) applied for people with stroke

**DOI:** 10.1186/s12913-025-12456-8

**Published:** 2025-03-04

**Authors:** Maria Larsson-Lund, Ida-Maria Barcheus, Maria Ranner, Irene Vikman, Lars Jacobsson, Eva Månsson Lexell

**Affiliations:** 1 Occupational Therapy, Department of Health, Education and Technology, Luleå University of Technology, Luleå, Sweden; 2 Department of Health Sciences, Lund University, Lund, Sweden; 3 Department of Neurology, Rehabilitation Medicine, Memory Disorders, and Geriatrics, SkåNe University Hospital, Lund-Malmö, Sweden; 4 Department of Rehabilitation Medicine, Sunderby Hospital, Luleå, Sweden

**Keywords:** Stroke, Daily activities, Self-management, Lifestyle intervention, Internet-based rehabilitation, Tele-rehabilitation, Digital e-health solutions, Occupational therapy

## Abstract

**Background:**

To enable people with stroke to achieve an active everyday life under altered conditions, the development of self-management programs is essential to facilitate the process of change that individuals must undergo. To improve access to self-management, internet-based solutions have been proposed. The aim of this study was to evaluate the feasibility of a novel internet-based intervention, “Strategies for Empowering activities in Everyday Life” (SEE, version 1.0), for clients with stroke.

**Methods:**

This feasibility study had a preposttest design without a control group and utilized a mixed-method approach. Data were collected through study-specific forms, outcome assessments, interviews, and field notes. Descriptive statistics and content analysis were subsequently applied.

**Results:**

The study involved fifteen clients and staff at clinics in a hospital-based open-care rehabilitation setting. The results indicate that SEE is feasible for clients with stroke. When adopted as expected, SEE has the potential to empower self-management and enhance engagement, balance, and values in everyday activities. The study also indicates that SEE is feasible in terms of adherent delivery of dosage, acceptability, and value, as perceived by clients, occupational therapists, and clinic managers. However, adjustments are needed in the study design, in terms of recruitment strategies, the selection of assessor-based outcome assessment, and the evaluation of adherence. Additionally, the educational program for professionals should be enhanced to better support the implementation of SEE.

**Conclusion:**

After the study design, intervention, and educational program are refined, SEE can be prepared for a pilot randomized controlled trial.

**Trial registration:**

clinicaltrails.gov NCT04588116, date of registration: 8th October 2020.

**Supplementary Information:**

The online version contains supplementary material available at 10.1186/s12913-025-12456-8.

## Background

Stroke is one of the leading causes of lifelong disability worldwide [1]. People who have experienced a stroke often face unmet long-term needs related to body functions, daily activities, and social participation [2, 3]. Following a stroke, people often need to manage feelings of loss and gradually regain control and agency in their everyday lives [4]. Thus, addressing how they can manage long-term consequences to enable an active and healthy everyday life is crucial in stroke rehabilitation. Maintaining an active everyday life can be complex because it involves a satisfying distribution and a healthy balance of various daily activities, which take place in different locations in the community and include interactions with other people [5]. In this context, people need to be empowered to optimize their self-management skills to handle day-to-day challenges in relation to their new capacity and life situation.

The availability of stroke rehabilitation services from a long-term perspective is frequently limited [6, 7], with the support received often emphasizing functional recovery rather than addressing everyday life management [7–11]. Clients express a need for strategies that extend beyond traditional medical management to live well on new terms. Therefore, supporting self-management behaviours, such as analysing demands and employing a variety of interconnected strategies on a daily basis, is crucial [12, 13]. Thus, research indicates a growing need for more person-centred self-management support tailored to each person’s everyday life [13]. In line with this, the action plan for stroke in Europe [7] emphasises the necessity of developing and evaluating self-management programmes and exploring how digital services can be resources.

In response to these needs, an internet-based person-centred intervention, “Strategies for Empowering activities in Everyday Life” (SEE, version 1.0), was developed to support peoples’ self-management in everyday life [14]. In SEE, people explore how their daily activities impact each other and whether they are engaged in activities in a balanced and healthy manner. They learn to self-analyse and reflect on their engagement patterns, distribution of daily activities and overall well-being. Additionally, they acquire self-initiated management strategies for an active everyday life that promotes sustainable health [5, 14]. SEE thereby guides people through the change process necessary to recreate an active everyday life under new conditions.

The first version of SEE was developed in several steps, including a review of current evidence and the establishment of program theory and cocreation of the prototype [14]. In line with how new interventions should be developed and evaluated [15–17], a first test of SEE with users—both clients and occupational therapists (OTs) [18]—was performed to refine content and delivery. Moving to the feasibility phase [15, 19–21], uncertainties related to study design and intervention needed to be evaluated to inform the subsequent steps in evaluating SEE and guide refinement as needed.

The aim of the study was therefore to evaluate the feasibility of the internet-based intervention SEE, with a focus on study design and the intervention for people after stroke. Specifically, this feasibility study evaluated the following:

- Recruitment, retention/dropout, and resulting sampling characteristics.
- Adherence to the delivery of SEE.
- The outcome assessments’ suitability to detect changes at 4 and 12 months after SEE, as well as their acceptability.
- The acceptability and value of SEE.

## Method

### Study design

This feasibility study had a preposttest design without a control group and utilized a mixed-method design that included the collection and analysis of both quantitative and qualitative data. This design is guided by the Medical Research Council (MRC) guidelines [15] and related recommendations [19–21] for how complex interventions are evaluated from different aspects in the feasibility phase. The study followed a study protocol [5]. This study is part of a larger feasibility project that includes three studies. Two qualitative studies focused on the experiences of those who provided and received SEE have been reported elsewhere [22, 23]. This study was approved by the Swedish Ethical Review Authority (Dnr 2019–04993; 2021–03409; 2022-00519-02).

### Study settings

The study involved four hospital-based open rehabilitation clinics in northern Sweden. The SEE was delivered through the “support and treatment platform” within the Swedish Healthcare Guide; 1177.se and through platforms for video-meetings.

### Eligibility criteria for the inclusion of clients

The inclusion criteria for clients living in the catchment area of hospitals were as follows: (a) 18–75 years of age; (b) 6–12 months after the stroke; (c) moderate disability or good recovery after the stroke (level 5–8), as measured by the Glasgow Outcome Scale-Extended (GOSE) [24]; (d) discharged from team-based rehabilitation at the hospital or home/day care; (e) access to and ability to use a computer, internet and e-ID; and (f) being motivated for a process of change in daily activities. The motivation was investigated using three different questions [25] focusing on whether daily activities were a problem. whether they wanted a change and were currently ready to implement a change. The exclusion criteria were (g) depression as measured by the Hospital Anxiety and Depression Scale [26]; (h) other conditions or diseases that impact daily activities; and (i) other conditions such as aphasia or diseases that may affect informed consent and participation in the data collection and intervention. Three rehabilitation clinics included clients who had a stroke between April 2020 and December 2022, whereas a fourth rehabilitation clinic invited clients who had a stroke between April 2020 and December 2021.

After the recruitment process started, inclusion criterion b was amended twice. The 6–12-month time frame was established on the basis of the expectation that clients’ physical recovery would have reached a plateau. During this period, restrictions in everyday life often persist, and the need to manage these limitations becomes evident for clients [27–29]. However, the rehabilitation clinics suggested that clients’ readiness and need for SEE could arise even after twelve months. Consequently, the inclusion criteria for SEE were adjusted to 6–24 months in 2021. The criteria were further modified in 2022 to include clients earlier after stroke, expanding the criteria to 3–24 months.

### Recruitment procedures

Clients were enrolled from November 2020 to December 2022 to obtain a sample that could provide enough information about the feasibility aspects investigated [21]. From a database, the four participating rehabilitation clinics identified stroke clients during the study period. Potential clients were then screened against the inclusion and exclusion criteria, and those who fulfilled the criteria according to their clinical records (criteria a, b, c, d, h) received an information letter about the study. Potential clients were also asked to respond if they were interested in participating and wanted to receive more information about the study or if they were not interested in participating. If no response had been received within 2 weeks, the clinic contacted the potential client by telephone to ensure that no one was missed. Interested clients provided their contact information, which was passed on to the researchers. The researchers subsequently conducted telephone calls to describe the study, address questions, and obtain verbal consent. Written informed consent forms were sent, signed, and returned, and a video meeting confirmed the remaining inclusion criteria (c, e, f, g, i). Throughout their participation in the study, the researchers checked that their informed consent remained.

The rehabilitation clinic that participated in the initial SEE test during the development phase [18] was invited in 2020, and three other rehabilitation clinics received invitations in 2021. The recruitment pool of staff included OTs and their managers at the clinics who chose to deliver SEE. The OTs and their managers received verbal and written information about the study, and their verbal and written informed consent was obtained and, thereafter, asked for throughout the study period. Information meetings about project progress and results were conducted four times yearly during the study period with all involved staff at the rehabilitation clinics.

### Internet-based intervention SEE 1.0

Internet-based intervention SEE comprises three parts: (i) a web program in 1177 combined with digital video meetings and client materials, (ii) an internet-based educational program for OTs, and (iii) an intervention guide and materials for OTs [5, 14]. The program theory of SEE integrates models of occupational therapy, person-centeredness, motivation theories, self-management, rehabilitation, flipped-classroom pedagogy, and evidence of client changes in everyday life, as well as the design of internet-based interventions. The contribution of these components and how they are combined in the client intervention, the design of the education program, and the intervention guide of SEE are described in detail elsewhere [5].

**Fig. 1:**
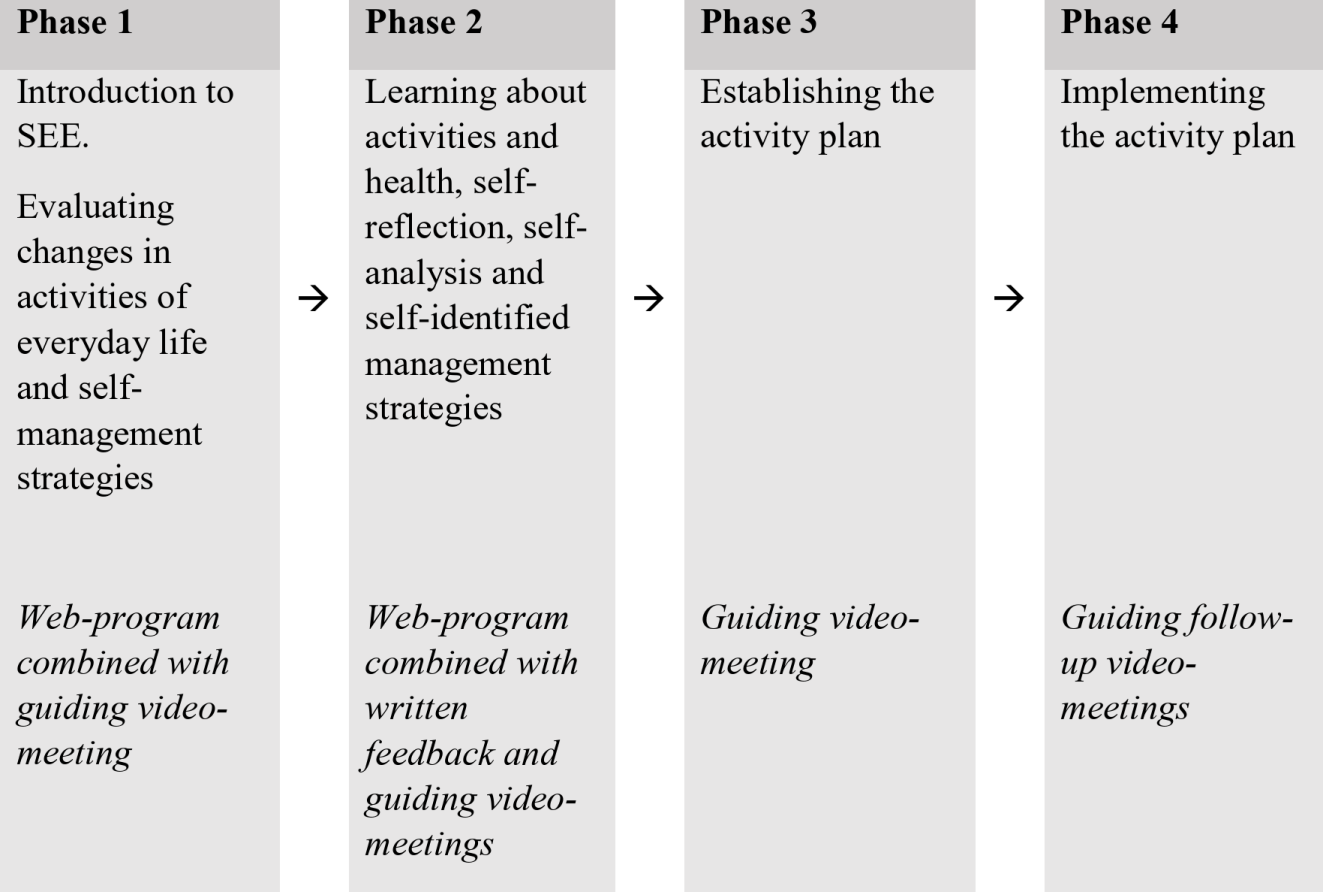
Phases of the SEE intervention process

The SEE intervention process spans four phases (Fig. 1), empowering clients to self-analyse everyday activities and develop self-initiated management strategies [14]. This enables them to become proactive in preventing and managing daily challenges over time. In the initial phase, clients engage in self-analysis of their activities and self-initiated management strategies. This involves assessments and reflective dialogues together with the OT during video meetings. The second phase includes web modules with educational video clips and related assignments on a health care platform. Additionally, clients participate in in-depth, online dialogues with OTs. In the third phase, clients collaborate with OTs to create an activity plan, focusing on person-centred goals and related management strategies. These three initial phases are expected to last up to four weeks. Finally, during the fourth phase (lasting 2–4 months or more), clients implement the plan with tailored guidance from the OT via video meetings until their goals are achieved.

The SEE educational program, intervention guide, and related materials were available for self-tutorials on an online learning platform to train OTs in delivering SEE [14]. These resources, combined with subsequent supervising workshops, aimed to ensure the adherence and consistency of SEE delivery. The educational program includes recorded videos covering SEE’s background, purpose, and how evidence, models, and tools are integrated in the intervention process based on the program theory. The intervention guide for OTs provides detailed information about SEE goals and the intervention process, including step-by-step guidance for each module in SEE. The intervention guide describes the module themes, content, client assignments, and how the OT, in a person-centered and motivating manner, is expected to guide the client. By empowering clients to self-reflect and analyse everyday life, they are supported in adopting management strategies and engaging in activities. The changes implemented in activities are continually monitored and reflected upon to achieve sustainable change. The OTs are provided with detailed guidance on conducting video dialogues and providing written feedback in the modules, including reflective questions to address in dialogues with clients and concrete suggestions on how to reply in a confirmatory and encouraging manner to support further self-reflection. Additionally, OTs could request supervision from the research group.

### Data collection

Feasibility was assessed using recruitment, retention, intervention adherence, acceptability, and value. Data were collected using several methods on different occasions (i-iv) (Table 1).

**Table 1:** Overview of the data collection methods used in the feasibility evaluation of SEE 1.0

Evaluation aspects	Data	(i) Baseline	(ii) During intervention	(iii-iv) Follow-up, four-month (iii), twelve months(iv)		(v) Afterstudy period
*Background data of clients*:	Sociodemo- graphic data, MFS^a^, HADS^b^, GOSE^c^	X				
*Recruitment and retention*	Registration forms completed by the clinic and researchers	X	X		X	
*Adherence*,* clients and OTs*	Field notes completed by researchers	X	X		X	
*Suitability and acceptability of outcome assessments*,* clients*	POES^d^, OBQ-11^e^, OVal-pd^f^, WAI^g^, LiSat-11^h^, GSE^i^	X			X	
*Acceptability*,* value and unintended harms*,* clients*	Study-specific form, self-report				X	
*Adherence and unintended harms*,* OTs*	Registration forms, implementation of SEE for each client after each module, completed by OTs		X			
*Acceptability and value*,* staff*	Structured interview in group with OTs Semi-structured interview in group with clinic managers					X X

^a^Mental Fatigue Scale (MFS)

^b^Hospital Anxiety and Depression Scale (HADS)

^c^Glasgow Outcome Scale Extended (GOSE)

^d^Profile of occupational engagement in people with severe mental illness (POES)

^e^Occupational Balance Questionnaire (OBQ-11)

^f^Concrete occupational value (OVal-pd)

^g^Work Ability Index (WAI)

^h^Satisfaction with life (Lisat-11)

^i^General self-efficacy (GSE)

#### Recruitment and retention of clinics, staff and clients

Recruitment and retention of clinics, staff and clientsThe clinics and their staff (OTs and their managers), who either accepted or declined participation, were documented. For the participating staff, information on sex, profession, and length of professional experience was obtained. In the registration forms for recruitment, the number of potential clients who met the inclusion and exclusion criteria, as well as those who chose to participate, declined or could not be reached, were recorded. Furthermore, those who dropped out during the baseline evaluation or during the SEE modules were recorded. The sociodemographic characteristics of the clients were collected at baseline, along with assessments of disability severity, fatigue, and anxiety/depression using the Glasgow Outcome Scale-Extended (GOSE) [24], Mental Fatigue Scale (MFS) [30] and Hospital Anxiety and Depression Scale (HADS) [26].

#### Adherence to the delivery

Researchers took fieldnotes after every meeting with both groups of participants (clients and OTs). These notes included information about any deviations from the intervention guide that could impact the outcome of SEE. Adherence to the SEE program’s delivery and identification of unintended harms were further assessed via study-specific feasibility registration forms (Appendix 1) completed by OTs for each client after every module or follow-up session during the intervention process. The feasibility registration forms included both structured questions (with checkboxes) and open-ended questions and were developed by the researchers for use only in this study. The structured questions focused on delivery adherence to the SEE intervention process for each client, as well as the adequacy of support provided by the SEE intervention guide. The open-ended questions aimed to capture fieldnotes related to aspects of fesaibility of the intervention process. The number of complete registration forms for each client was counted.

#### Suitability and acceptability of the outcome assessments

The selected standardized outcome assessments were evaluated on the basis of their suitability to detect changes after SEE from baseline to four and twelve months. Additionally, the acceptability of the assessments was assessed by number of fully completed assessments. The selected assessment reflects the core of SEE. The Profiles of Occupational Engagement Profile of Occupational Engagement in people with Severe mental illness (POES) [31] assesses the level of engagement in occupations by a summed score between 9 and 36 points. The Occupational Balance Questionnaire (OBQ-11) [32] assesses the level of having the right mix and variation of activities, which results in a summed score between 0 and 33. The Occupational Value predefined (OVal-Pd) [33] summarises different values present in activities in a general score (ranging between 18 and 72) and sub-scores. Three questions from the Life Satisfaction Questionnaire (Lisat-11) [34, 35] were used to assess satisfaction with life as a whole as well as physical and mental health. The items are rated on an ordinal scale ranging from 1 to 6, and a higher score reflects a higher level of satisfaction. Self-management was assessed with the General Self-Efficacy Scale (GSE) [36, 37] with a summed score ranging from 10 to 40. Workability, both actual and perceived, was evaluated for working-age clients. The Work Ability Index (WAI) [38] assesses perceived work ability on a ten-point ordinal scale. A higher score in all assessments indicates better results. The self-reports (OBQ-11, OVal-Pd, Lisat-11, GSE, WAI) were filled in by the clients and returned by mail to researchers at baseline, four and twelve months. The assessor for the interview-based POES assessment was changed between the baseline assessment and the two follow-up assessments, with the second and third authors taking on this role. The client’s baseline assessment results (POES, OBQ-11, OVal-Pd), as part of phase one in SEE, were reported to OTs delivering SEE to a particular client, following a specific protocol.

#### Acceptability and value

The acceptability and value of SEE were assessed at the clients’ four-month follow-up via a study-specific questionnaire developed by the research team (Appendix 2 and 3). Clients rated acceptability in terms of satisfaction with various components of SEE (Appendix 2) and the value of the intended benefits of SEE (Appendix 3). Ratings were provided on a four-point ordinal scale ranging from “not at all satisfied” to “very satisfied” for acceptability and from “do not agree at all” to “strongly agree” for perceived value. In addition, an open-ended question covered unintended harms.

To explore the acceptability and value of SEE’s educational program, intervention guide and intervention process (dosage, content, logical order) from the deliverers’ perspective, a semi-structured group interview with OTs was preformed based on an interview guide (Appendix 4). The interview, which were conducted by the first and second authors after the study period, were audio recorded and transcribed verbatim.

Semi-structured group interviews [39] with clinical managers of those who delivered SEE were conducted at the end of the study period to explore the acceptability and value of SEE from an organizational perspective. The interview guide (Appendix 5) covered feasibility and acceptability aspects in various contexts, including the possibility of implementing SEE within their own organization or other organizational contexts. The interviews were carried out by the first and second authors and were audio recorded and transcribed verbatim.

### Analysis

Descriptive statistics were employed to analyse recruitment and retention, sample characteristics, and forms related to adherence, acceptability, and value. Descriptive statistics were used to analyse pre- to posttest changes in the outcome assessments POES, OBQ, OVal- PD, LiSat-11, GSE and WAI at baseline and at the four- and 12-month follow-ups.

The adherence to the delivery of SEE, as recorded in notes in registration forms and in researchers’ fieldnotes, was analysed on the basis of content [40] by the first, second and last authors. The answers to the structured questions about the acceptability and value of SEE from the OT’s perspective were also analysed in terms of content by the first and second authors. The transcribed group interviews with OTs regarding the acceptability and value of SEE were analysed via directed content analysis [41]. This approach for content analysis is deductively guided by a structure such as theory, key concepts or variables. When analysing the clinical managers’ group interviews, the concepts of feasibility, acceptability and value, together with implementation, guided the analysis. The authors read the transcripts to understand the content, assigned codes to text with similar meanings, grouped codes on the basis of predefined concepts, and conceptualized categories. In the final step, the transcripts were reviewed in relation to the evolving results. The second author conducted the main analysis, and the credibility of the evolving results in the analysis steps was confirmed by the first author.

## Results

First, the study samples will be presented, followed by the feasibility of SEE in four sections: (1) Recruitment and retention of clinics, staff, and clients; (2) adherence to delivery; (3) suitability and acceptability of outcome assessments; and (4) acceptability and value.

### Participants

All the included clients reported good recovery after their stroke according to the GOSE [24], and Table 2 shows the characteristics of the included participants. The clients were divided in two groups based on intervention adherence, that will be further explained in a coming section. As the client group is small, differences in demographics should be considered with caution, but Group B (who received SEE with deviation, SEE dev.) tends to have participants with a longer time since injury than does Group A (who received SEE per protocol, SEE PP). Only one client received other types of rehabilitation during the follow-up period.

**Table 2:** Characteristics of included clients with stroke, *n* = 15

	Total *n*:15	Group A *n*:6	Group B *n*:6	Drop out *n*:3
Female/Male	6/9	4/2	2/4	0/3
Age, Median, IQR, (range)	64, 15, (35–73)	61, 15 (43–71)	65, 34 (35–73)	67 (65–68)
Time since injury, months, median; IQR, (min–max)	8; 7 (3–25)	6; 6 (3–10)	13; 15 (6–25)	8 (6–18)
*Education level:*				
Primary school/Upper secondary school/University	1/7/7	0/2/4	1/4/1	0/1/2
Working Full-time/part-time/Retired	2/6/7	1/3/2	1/2/3	0/1/2
*Living condition:*				
Married/cohabiting and/or with children	12	5	5	2
Living alone	1	1	1	1
Lives in own house/apartment	6/9	4/2	1/5	1/2
Driving car, yes/no	9/6	4/2	4/2	1/2
*Rehabilitation after acute care*:				
No follow-up	2	1		1
Follow-up calls	5	2	3	
Outpatient rehabilitation	7	3	2	2
Inpatient rehabilitation	1		1	
*Rehabilitation during follow-up of SEE*, inpatient rehabilitation			1	
*Presence of anxiety/depression*^*a*^:				
Mild to moderate anxiety		1	2	0
Depressive		0	0	0
MFS^b^, Median; IQR, (min–max)	10,5; 9 (0,5–29,5)	13,75; 12 (1,5–21)	9,75; 15 (0,5–29,5)	7,5 (6,5–9,5)
Total number of participants with fatigue	7	4	2	1

^a^ Hospital Anxiety and Depression Scale (HADS)

^b^ Mental Fatigue Scale (MFS), min–max score: 0–44 p, with a cut-off score of 10.5 where fatigue should be considered/affects activity

Four OTs (three women and one man) from the two rehabilitation clinics who delivered SEE were invited and agreed to participate. These OTs had professional experience ranging from ten to twenty-four years and as much clinical experience with stroke clients. Each OT delivered SEE to two to six clients. Furthermore, three managers from the same rehabilitation clinics as the OTs were invited and agreed to participate. All managers were women, two with an occupational therapy background and one as a physiotherapist. Their management experience ranged from 0.5 to 11.5 years, and within the organizations, where SEE was delivered for 0.5 to 9 years.

### Recruitment and retention of clinics, staff and clients

Owing to variations in the timing of participation, organizational changes, and resource availability, the type of involvement of clinics varies. They also entered and concluded their involvement at different times. The rehabilitation clinic involved in the development phase of SEE also chose to continue their participation in this feasibility study, and the three additional invited rehabilitation clinics expressed an interest in enrolling clients, with two planning to deliver SEE. Thus, one clinic enrolled clients to another clinic who delivered SEE. One of the clinics that planned to deliver SEE withdrew after a year, as the effort to recruit clients had been fruitless and their resources were directed towards managing the COVID-19 pandemic.

**Fig. 2:**
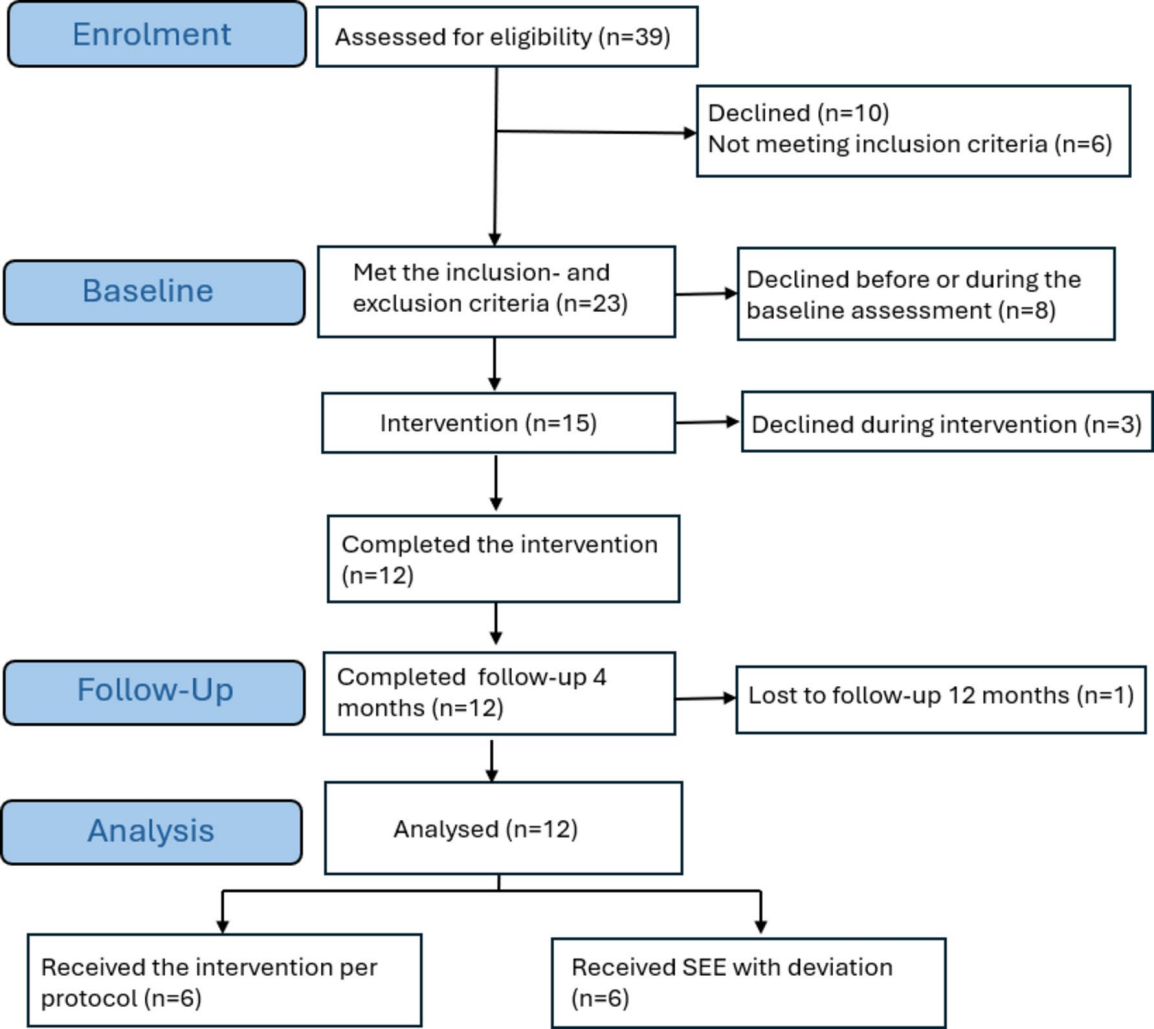
Flowchart of included participants and those who completed intervention, follow-up, and were analysed

Of the 179 people who received written information about the study from the rehabilitation clinics, 39 showed interest and agreed that their contact details be passed to the research team. The recruitment rate from eligible assessment to intervention start was 41% (Fig. 2). During the first telephone call and the first e-meeting when the inclusion screening was performed, ten people (26%) declined or could not be reached, and six people (15%) did not meet the inclusion criteria (three had a severe disability according to the GOSE, two had a lack of technical experience/equipment, and one had recently experienced a new stroke). Twenty-three people fulfilled the inclusion and exclusion criteria to participate in the study, but eight people (20%) declined prior to or during the baseline assessment. Finally, 15 clients agreed to participate and started the intervention. The retention rate for the intervention was 12 people (80%) as three people dropped out (one entered inpatient rehabilitation, and two declined). The retention rate for the 4 month follow- up was 100%, and for 12-month follow- up 92% as one participant could not be reached. All the personnel retained their participation throughout the study.

### Adherence to the delivery

The intervention adherence was insufficient (50%). The results revealed that six clients received SEE per protocol (PP) (Group A), and six clients received SEE with deviation (dev.) (Group B), as their change process did not start or evolve as expected. The researchers’ field notes, supported by the registration forms for adherence, revealed that the different processes that evolved were related to a combination of factors. For example, clients focused on impairments, had a varied level of motivation to change, and had difficulties taking on the content of SEE and taking responsibility for implementing it in their life situations. Additionally, OTs were new to and inexperienced in delivering the program to facilitate clients’ change process. This meant that all clients in Group B’s participation in establishing their activity plan was limited, as the clients were not able to identify necessary changes in everyday life.

The adherence rate for the delivery of the dosage and order of the modules in three initial phases of SEE, in accordance with the intervention guide, was 100% (*n* = 11 clients, forms for one client were missing). For six of the clients (50%) (all in Group B, SEE dev), the modules were delivered with longer or shorter time intervals than those recommended in the guide. The time deviation/delay varied between six and 14 days. In the fourth phase of SEE, the number of follow-up meetings that, according to the intervention guide, are individually tailored varies between 0 and 6 follow-ups (Group A: 1–6 *m*: 3 follow-ups; Group B: 0–5 *m*: 2 follow-ups). Consequently, 10 clients (91%) received the expected follow-up, and one client (in Group B) did not receive any follow-up, i.e., a deviation from the intervention guide. For the clients in Group A (SEE PP), SEE lasted between 1 and 10 months (*m*: 6 months), whereas for the clients in Group B (SEE dev) (*n* = 5), it lasted between 1 and 6 months (*m*: 4.5 months). No deviance from the planned content of the sessions, in relation to the intervention guide, was reported by the OTs. Additionally, the OTs reported that the guide provided them enough support to accomplish each module for each client.

Unintended harms were reported in 12 forms due to technology difficulties (in the video-meeting at ten occasions for clients and one occasion for an OT and for one client difficulty with 1177).

The evaluation of the acceptability of the registration forms showed that they were returned for 11 of 12 clients (92%); for one client (Group B), no form was provided. In total, 98 forms were accessible. The response rate of the forms was 92% as eight forms were missing compared with the delivered sessions according to fieldnotes: five forms for one client, two for one client and one form for another. The missing forms were mostly related to the follow-up sessions. All the questions in the forms with checkboxes or fill-in fields related to adherence were completed for all the clients (100%), but the open-ended questions provided limited or no information, making it difficult to analyse the responses.

### The outcome assessments

The analysis of the suitability of the outcome assessment showed that they identified changes within and between groups (Table 3). Group A, which finalized SEE per protocol, showed a positive change in all outcome assessments (occupational engagement, occupational balance, occupational value, satisfaction, self-efficacy, and work ability) from baseline to the both follow-ups. Group B, which finalized SEE with deviation, showed no change to a minor negative change for all assessment tools, except for POES. However, in total, the clients received a maximum score on the POES at 14 of 36 measurement points (39%) (one client at baseline, six at 4 months, and seven at the 12-month follow-up).

The response rate of the outcome assessment, reflecting acceptability, was high. The response rate on all the outcome assessments at baseline and at the four-month follow-up (POES, OBQ-11, OVal-PD, GSE, LiSat-11, WAI) was (100%). At the twelve-month follow-up, ten out of the eleven remaining clients returned all the assessments, one client (Group B) completed all the assessments with the exception of POES (98% of the total number of assessments). All of the returned assessments were complete, with one exception: GSE had one missing item for one client (Table 3).

**Table 3:** Results of the outcome assessments from baseline to follow-up at 4 and 12 months for all clients and per group (Group A, completed SEE per protocol; group B, completed SEE with deviation)

	All clients				Group A			Group B		
Months	0		4	12	0	4	12	0	4	12
Number of responders	*N* =12		*N* =12	*N* =11	*N* = 6	*N* = 6	*N* = 6	*N* = 6	*N* = 6	*N* = 5
	*Median; IQR (min–max)*				*Median; IQR (min–max)*			*Median; IQR (min–max)*		
Profile of occupational engagement, score: 1–36 p	29,5; 16 (20–36)	35,5; 5 (21–36)		36^a,b^; 6 (21–36)	27,5; 7 (26–36)	36; 4 (30–36)	36; 4 (29–36)	30,5; 12 (20–35)	31,5; 12 (21–36)	34,5^a,b^; 12 (21–36)
Occupational balance, score: 0–33 p	20; 9 (11–26)	22; 13 (9–33)		22; 10 (8–30)	19,5; 9 (13–25)	25; 11 (19–33)	23,5; 7 (16–30)	20; 10 (11–26)	18; 14 (9–27)	17; 10 (8–25)
Occupational values, score: 1–72 p	44; 16 (32–58)	48; 13 (36–65)		52; 9 (36–60)	43; 11 (38–57)	50,5; 13 (41–60)	53; 6 (49–60)	47; 20 (32–58)	42; 21 (36–65)	46; 14 (36–55)
Self-efficacy score: 10–40 p	30; 10 (22–36)	33^c^; 11 (22–39)		29^d^; 11 (22–38)	28,5; 11 (23–36)	35^c^; 11 (22–39)	32^d^; 11 (25–38)	31; 10 (22–34)	30,5; 10 (24–36)	29; 5 (22–30)
Satisfaction with;										
Life as whole	5 (3–6)	5 (2–6)		5 (3–6)	4 (3–5)	5 (4–5)	5 (5–6)	5 (3–6)	4 (2–6)	4 (3–4)
Somatic health	4 (2–6)	4 (3–6)		4 (2–6)	4 (2–4)	4 (3–5)	4 (3–6)	4 (2–6)	4 (3–6)	4 (2–6)
Psychological health score: 1–6 p	4,5 (3–6)	5 (3–6)		5 (2–6)	4 (3–6)	5 (4–6)	5 (4–6)	5 (3–6)	4 (3–6)	5 (2–6)
Work ability^e^ score: 0–10 p	6 (3–10)	6,5 (2–10)		7 (3–9)	7 (3–8)	7 (5–9)	8 (5–9)	5 (3–10)	6 (2–10)	5 (3–7)

^a^ One client in Group B was not reached for follow-up

^b^ Not completed by a client in Group B

^c^ Not completed by a client in Group A as the tool was not sent out due to a mistake

^d^ One item was not answered by a client in Group A. The item was given a number based on a median of the other answers to enable summation

^e^ Eight clients received WAI, and four clients in each group worked to some extent

### Acceptability and value

Overall, the clients rated the acceptability of SEE components as satisfied or very satisfied (Table 4). In general, the clients agreed or strongly agreed with the statements of the value of SEE (Table 5). Overall, both the acceptability and value of SEE were rated slightly higher in Group A (SEE PP) than in Group B (SEE dev). No harm was reported in the open-ended questions by the clients.

**Table 4:** Client acceptance of SEE at the four-month follow-up

Acceptability with the following component*:	Total *n*:12	Group A *n*:6	Group B *n*:6
	Median (min–max)		
SEE as a whole	3,5 (2–4)	3,5 (3–4)	3,5 (2–4)
The focus of SEE	4 (3–4)	4 (3–4)	3 (3–4)
The distance format	4 (2–4)	4 (3–4)	3,5 (2–4)
The combination of the web- program with online meetings	4 (2–4)	4 (3–4)	3,5 (2–4)
The educational clips in the web- program	3 (2–4)	3,5 (3–4)	3 (2–4)
The reflection tasks and self-assessment forms in the web-program	3 (2–4)	3 (3–4)	3 (2–4)
The support of the OT	4 (3–4)	4 (4–4)	4 (3–4)
The number of online meetings with the OT	3,5 (3–4)	4 (3–4)	3 (3–4)
The content of your activity plan	4 (2–4)	4 (3–4)	3 (2–4)
Follow-up support by the OT during the realization of the content of the activity plan	4 (3–4)	4 (4–4)	3,5 (3–4)
The number of meetings with the OT during the realization of your activity plan	4 (3–4)	4 (3–4)	3 (3–4)
Your own effort during SEE	3 (2–4)	3 (3–4)	3 (2–4)
The result you achieved from SEE	3 (2–4)	3 (3–4)	3 (2–4)

*Items were answered on a four-point scale from 1 = not at all satisfied, 2= partly satisfied, 3 = satisfied, and 4 = very satisfied

**Table 5:** Client perceived value of SEE at the four-month follow-up

Statement on the value of SEE*	Total *n*:12	Group A *n*:6	Group B *n*:6
	Median (min–max)		
Have increased my knowledge of the importance of activities in everyday life to feel good and experience health	3 (2–4)	4 (3–4)	3 (2–4)
Has given me new insights into my pattern of activities in everyday life and my balance in activities and how they affect my well-being	3 (2–4)	4 (3–4)	3 (2–4)
Has helped me to “see” my activities in everyday life in a new way	3 (2–4)	4 (3–4)	2,5 (2–4)
Has given me insight into how I can change activities in my everyday life to achieve an active life under my new conditions	3 (2–4)	3,5 (3–4)	3 (2–4)
Has given me strategies that I can use/have used to change my everyday life	3 (2–4)	3,5 (3–4)	3 (2–4)
Has helped me take an active role in changing my life situation	3 (2–4)	4 (3–4)	3 (2–4)
Has given me an increased readiness to take on everyday challenges in the future	3 (1–4)	4 (3–4)	2,50 (1–4)
Has helped me in my change process in my new life situation	3 (1–4)	4 (3–4)	3 (1–4)

*Items were answered on a four-point scale from 1 = strongly disagree, 2 = agree to some extent, 3 = agree, and 4 = strongly agree

The OTs’ experiences demonstrated that both the SEE educational program and the intervention guide were acceptable and valuable, as these resources provided the necessary training and support for delivering SEE, with no missing components reported. The content of the intervention guide was perceived as solid, easy to follow, and adaptable to each client. The OTs faced challenges during the initial implementation of SEE, needing time and experience to fully grasp the whole concept. As a result, they dedicated extra time to revisiting the educational program, intervention guide, and client materials. This reinforcement and learning process was particularly necessary when long intervals occurred between new clients. They also found it challenging to document their new way of working in client records and communicate it in an understandable manner to other professionals.

The OTs reported that the SEE intervention process was acceptable, as the content and dosage of the modules, including the session dosage for digital meetings, were relevant for supporting clients’ change processes. The OTs observed that the combination of the clients’ preparations through the web program and the guiding dialogues during their meetings fostered client engagement and responsibility for implementing changes. Therefore, they emphasised importance of encouraging clients’ preparations for meetings. The OTs did not report any missing content, and the order of the modules was logical.

The analysis of the clinical managers’ experiences formed two *categories: SEE was feasible and a valuable step in developing rehabilitation services*, and *the implementation of SEE involved organizational changes.*

*SEE was feasible and a valuable step in developing rehabilitation services.* The managers experienced that SEE was valuable in their organizations and believed that it had the potential to improve and advance rehabilitation services since there was a lack of similar initiatives in health care. They reasoned that the time the OTs had to allocate to learn the program was similar to that of other programs and that implementing SEE also had the potential to save resources. This is reflected in the following quote: *“If SEE turns out to be effective*,* it is a method that you should adopt […] then it is obvious that it is part of our mission*,* and we should keep up with what’s evidence-based.”* (Clinic managers [CM]. 2).

The managers believed that SEE was suitable for clients who can manage digital formats and clients who can be supported by OTs in taking responsibility for their own rehabilitation. Drawing from their experiences in implementing SEE and other rehabilitation services, the managers discussed the challenge of identifying motivated clients who prioritize investing the time necessary for successful rehabilitation, including SEE. They also emphasized the importance of receiving timely and coherent rehabilitation interventions. That is, interventions that reach the right people at the right time without interruptions due to holidays or other reasons are a challenge that is not unique to SEE. They added that SEE has the potential to meet the needs of several diagnostic groups with long-term disease. The implementation of SEE contributed to the development of occupational therapy by enhancing OT competencies in adopting new approaches and utilizing digital tools in an innovative manner. One of the managers said, *“I think it has been an incredible learning process for occupational therapists to use digital tools with clients because we have not done it before. I think it is a step in the right direction. […] it will be an important part of the future of occupational therapy” (CM. 1).*

The managers expressed that SEE could serve as a valuable tool, contributing to their ongoing work in establishing integrated and person-centred care. They highlighted that SEE’s digital format opens new opportunities for equitable and accessible care. For example, it facilitates the collaboration and sharing of OT resources between different organizations, especially during periods when it is difficult to recruit OTs.

*The implementation of SEE involves organizational changes.* The managers said that implementing SEE as part of their routine rehabilitation would require organizational changes. The managers discussed the need to review predefined rehabilitation focuses and durations of rehabilitation periods to offer more flexible and person-centred services aligned with SEE. This needs to involve a review of how each profession’s competencies can best serve the client. The managers emphasized that implementing SEE does not conflict with the team perspective in their organization, but it requires a shift in team practices. As one manager said, *offering good occupational therapy means looking at each person (client) individually. (…) It will be a challenge for us to get it right*,* because of the old*,* cemented way of working and schedules and… but it is all small things actually… so I think that it [SEE] is consistent with what we have felt [we need] since before” (CM. 1).*

The managers discussed that they wanted to offer internet-based rehabilitation alternatives and emphasized the need for their entire organization to refocus efforts on achieving sustainable changes in clients’ everyday lives over the long term. When comparing SEE’s impact to that of ordinary rehabilitation, one manager said, “*Being able to zoom out as an occupational therapist to guide and coach*,* the client can take more responsibility*,* a bit like [refer to another participant’s opinion] CM2 said that you should not come for 8 weeks for these 60-minute sessions and then go home and do as usual or as before. Here*,* [referring to SEE]*,* the process goes so much further because the clients are doing a different job. Therefore*,* because they are involved in the process in a different way*,* SEE is good from a person-centred perspective” (CM. 1).*

The managers described how their organizations were in a transition to integrate internet-based rehabilitation in practice. They emphasized that to achieve efficient implementation of new interventions such as SEE, professionals need education and supervision since this type of intervention requires a new way of approaching clients. On the basis of the positive experiences of SEE, the managers’ opinion was that the intervention could be implemented in many different contexts than their own, especially in outpatient care across different organizational levels, including primary care.

## Discussion

This study evaluated the feasibility of the internet-based intervention “Strategies for Empowering activities in Everyday Life” (SEE, version 1.0) for clients recovering from a stroke. Overall, the results indicate that SEE is feasible and, when adopted as expected, has the potential to improve occupational engagement, occupational value, occupational balance, and self-efficacy for stroke clients, with sustained improvements lasting one year after starting the intervention. Although the delivery of the SEE intervention process adhered to the module dosage for all clients, it deviated from the intervention guide in half of the cases (6 out of 12) for client- or occupational therapist-related reasons. For these clients, no changes were observed in the outcome assessments. Consequently, adherence to the intervention process and client outcome divided the clients into two groups. However, irrespective of adherence and outcome, all the clients reported high overall acceptability of the SEE design and various components. They also consistently rated SEE’s impact on self-management values in everyday life as high.

To improve adherence, the results suggest that the next version of SEE needs to incorporate multiple measures to reduce the number of non-responders. Being able to detect if clients are motivated and ready for a change is crucial during inclusion as well as throughout the intervention process. Furthermore, being able to identify whether SEE is timely and appropriate for a specific client early in a client’s rehabilitation process is important. This assessment can be guided by the transtheoretical model of behavioural change [25] and motivational interviewing [42] and is already included in the programme theory of SEE [14]. Moreover, enhancing the learning process by mastering the different components constituting the program theory of SEE while adhering to the prescribed content and delivery timeline is essential. By refining the educational program and the intervention guide, OTs can be empowered to coach client engagement and responsibility more effectively. To enhance clients’ ability to self-analyse their activities in everyday life and establish activity plans, adjustments to the SEEs intervention process, including web module content, are needed. As indicated, embracing SEE as intended can result in clients learning sustainable self-management skills, aligning with other non-internet-based occupational therapy interventions [43–45] that emphasize self-analysis and goal setting as therapeutic agents for change in daily life.

From the perspective of OTs and clinical managers, SEE—both the client intervention and the therapists’ educational program—were considered feasible, acceptable, and valuable. Their experiences of the effort to take on SEE, as it deviates from OTs’ ordinary work, also note the necessity of exhaustive education for OTs, especially in the context in which rehabilitation focuses foremost on functional recovery. Furthermore, the managers emphasized the need for organizational changes to implement new internet-based interventions with redesigned intervention processes, as in SEE. To facilitate the requested changes in health care in Sweden, aiming for a more proactive, person-centred, and integrated care approach [46, 47], the implementation of SEE could serve as a valuable tool for developing and expanding their rehabilitation services. These results suggest that the desired transformation in rehabilitation services may require various professional groups to adapt in different ways, fostering proactivity and person-centeredness within their respective areas of expertise and necessitating a new, more flexible approach to teamwork. Consequently, the integration and implementation of SEE in a rehabilitation context deserve attention in future research. This study indicates, in line with the results from two previous qualitative studies within the same feasibility project, that SEE has the potential to address self-management needs that are often overlooked after stroke [22, 23] and add value, currently missing in existing interventions [6, 7]. Consequently, SEE can provide OTs with a greater diversity of tools in their professional toolbox, enhancing opportunities to provide timely, person-centred support after a stroke. As SEE focuses on consequences in everyday life and not on the stroke per se, it can, in accordance with managers’ suggestions, be tested for other groups of clients in future research.

The feasibility evaluation of the study design reveals several areas for improvement. The recruitment of clients was anticipated to be challenging, given that no universally accepted strategies to overcome this challenge in intervention studies currently exist [48, 49]. However, the level of difficulty surprised us to some extent. In our pretest of SEE [18], which included only one clinic, approximately one-fifth of the clients who received the information letter agreed to participate. Additionally, our previous cross-sectional studies [50–52] involving people with acquired brain injury in the same geographical area identified needs related to engagement, value, and balance of activities in everyday life. Despite addressing these needs in SEE, expanding the research to more rehabilitation clinics, and having a long enrolment period, only 12 clients completed SEE in the present study. To increase the number of clients in future research, professionals could individually explain and recommend SEE to suitable clients. Providing personalized information and guidance might encourage more clients to participate. Clients may find it challenging to adopt a new internet-based intervention and may not fully understand how self-management programs can facilitate their everyday lives. Moreover, since many clients with stroke prefer to take part in research focused on regaining lost functions [53], identifying those in need of self-management and explaining its benefits within current rehabilitation services can be challenging. Other research [54] have highlighted the difficulty of providing information about new interventions that is understandable to clients. The recruitment target for the study [5] was not met, and the resulting sample had limited sociodemographic diversity (such as educational level) and included only those with good recovery. Despite this, the sample is still considered sufficient to inform adjustments of SEE and guide further feasibility research aimed at achieving a more diverse sample. In this first feasibility study of SEE, individuals with severe disabilities were excluded due to prior research indicating challenges with technology use [55]. Future research need to explore their potential for internet-based rehabilitation.

The assessment tools and the study-specific forms for acceptability and values were completed to a high extent (above 90%), indicating acceptability. However, it is possible that the number of assessment tools had a negative effect on the sample size, as eight participants withdrew before or at baseline. To mitigate this, clearer support for completing the assessments could be offered in future research. Furthermore, the assessor-based assessment POES [31, 56] exhibited a ceiling effect in approximately one-third of the assessments, rendering it unsuitable as an outcome measure. An alternative option is to replace it with the ‘Satisfaction with Daily Occupations’ (SDO) tool, which is sensitive to changes in everyday life [57]. Additionally, on the basis of the results from the two groups, it appears that the other outcome assessments (OBQ-11, OVal-PD, GSE-10, LiSat-11, and WAI) have the potential to detect changes after SEE. Nevertheless, the suitability of assessment tools and the potential outcomes of SEE need further exploration in future pilot randomized controlled trials (pilot-RCTs). The study-specific feasibility registration forms were in part unusable due to missing forms and incomplete fill-in fields. As the checkboxes were completed, an alternative approach could involve using forms exclusively with checkboxes or conducting structured interviews to address intervention adherence and consistency. Additionally, fieldnotes taken during researchers’ interactions with clients or OTs have proven valuable for understanding feasibility, adherence and consistency and will be important to continue with in forthcoming research.

As this was our first feasibility study of SEE and similar internet-based interventions in occupational therapy are lacking, no pre-specified progression criteria were set for the various feasibility aspects assessed. In line with discussions [17, 58] a more dynamic realist approach to feasibility can be beneficial rather than judging feasibility based on strict thresholds for progression to a future evaluation study. Therefore, the research group discussed the results of the various feasibility aspects individually and collectively, reaching mutual interpretations of feasibility as presented here. SEE deserves further feasibility research with a pilot-RCT design, allowing for the development of progression criteria for a potential future evaluation study, in accordance with recommendations for pilot-RCTs [59].

## Conclusion

These results indicate that SEE is feasible for clients with stroke. Adherence to the SEE intervention process divided clients into two groups: those who completed SEE per protocol and those who completed SEE with deviation. For the clients who completed SEE per protocol, the intervention showed the potential to enhance self-management, engagement, balance, and values in activities in everyday life. Deviations from the process resulted in no changes in outcome assessments. Regardless of the client group, SEE demonstrated feasibility in terms of dosage delivery, acceptability, and perceived value by clients, occupational therapists, and clinic managers. Further research is needed to assess adjustments of SEE components (the intervention process, the educational program, and the intervention guide) and study design improvements. The next step is implementing a pilot-RCT feasibility study for SEE.

## Supplementary Information

Supplementary Material 1.

Supplementary Material 2.

Supplementary Material 3.

Supplementary Material 4.

Supplementary Material 5.

## Data Availability

Owing to the Swedish Act for Ethical Review of Research on Humans and the Swedish Secrecy Act, the data are not publicly available. They are archived at Luleå University of Technology in accordance with Swedish laws and regulations. Requests to access the data can be sent to registrator@ltu.se.

## Abbreviations

GOSE: Glasgow Outcome Scale Extended
GSE: General self-efficacy scale, 10 items
HADS: Hospital Anxiety and Depression Scale
LiSat-11: Life Satisfaction questionnaire, 11 items
MFS: Mental Fatigue Scale
OBQ-11: Occupational Balance Questionnaire, 11 items
OTs: Occupational therapists
OVal-pd: Occupational Value pre-defined
POES: Profile of occupational engagement in people with severe mental illness
SEE: Strategies for Empowering activities in Everyday life
SDO: Satisfaction with Daily Occupations
WAI: Work Ability Index
