# Supplementary material for: A feasibility study of the internet-based intervention “Strategies for Empowering activities in Everyday life” (SEE 1.0) applied for people with stroke
Source: BMC Health Serv Res. 2025 Mar 4;25:330. doi: 10.1186/s12913-025-12456-8 (PMC11877923; doi:10.1186/s12913-025-12456-8)
Supplement: Supplementary file 1 — Supplementary Material 1. [file 12913_2025_12456_MOESM1_ESM.docx]

**Registration form for module 1-7 in SEE**

**Client no.: OT no. : Module no.: Module completed, date:**

Has the module been completed by the client in accordance with the given instructions?

⃝⃝ Yes ⃝ No

If no, what deviates?

Did the guide provide the support you needed in working with the module?

⃝⃝ Yes ⃝ No

If no, what was missing?

Were there any problems with the module? If so, how were they solved?

*Additional question related to the online meetings for modules 1, 4 and 7*

Time spent on the preparation of the dialogues/guiding session …. minutes

Time spent on the guiding session? .... minutes

Was the participant prepared for the dialogue? For example, did you need to help clarify their self-analysis and reflection? If so, what did you clarify and how?

Was there anything particularly challenging in the dialogue?

*Additional questions after module 7*

Did the participant complete (all) the modules in SEE within three weeks?

⃝⃝ Yes ⃝ No

If no, how long did it take to accomplish all modules? …. days

If no, what was the reason?

Was there any particular module or modules where the client’s reply was longer than three days?

⃝⃝ Yes ⃝ No

If yes, which module(s)? Please specify the module number(s):

Did the delay have any significance? Please specify

The occupational therapist’s (i.e., your own) reflection on SEE in the work with this particular client. For example: How did it work to establish a relationship and communicate digitally with this particular client? What are your experiences of using SEE as an occupational therapist with this particular client?"

**Registration form after module 7 in SEE**

**Client no.: OT no. : Follow-up date:**

The focus of the follow-up

⃝⃝ The activity plan ⃝ A specific measure, specify which one……….

Time spent on the preparation of the dialogues/guiding session: …. minutes

Time spent on the guiding session: …. minutes

Does the client follow the activity plan? Explain:

What support does the client need in the change process?

Does the activity plan need to be revised? If so, how?

Would you need any support? If yes, describe:

*Additional questions after SEE is completed for the client*

State which main/sub-goals from the activity plan the client has achieved.

If a goal or goals from the activity plan were not reached, describe them and explain the reason.

How do you assess the person's readiness to self-manage challenges in the future to maintain an active everyday life?

The occupational therapist’s (i.e., your own) reflection on SEE in the work with this particular client after the activity plan was established:
