# Supplementary material for: A feasibility study of the internet-based intervention “Strategies for Empowering activities in Everyday life” (SEE 1.0) applied for people with stroke
Source: BMC Health Serv Res. 2025 Mar 4;25:330. doi: 10.1186/s12913-025-12456-8 (PMC11877923; doi:10.1186/s12913-025-12456-8)
Supplement: Supplementary file 2 — Supplementary Material 2. [file 12913_2025_12456_MOESM2_ESM.docx]

**Satisfaction with “Strategies for Empowering Activities in Everyday Life”**

**(SEE)**

Mark your answer with a cross in the appropriate box

|  |  |  |  |  |
| --- | --- | --- | --- | --- |
| **How satisfied are you with:** | **Not at all satisfied** | **Partly satisfied** | **Satisfied** | **Very satisfied** |
| SEE as a whole |  |  |  |  |
| The focus of SEE |  |  |  |  |
| The distance/internet-based format |  |  |  |  |
| The combination of the web- program with online meetings |  |  |  |  |
| The educational clips in the web- program |  |  |  |  |
| The reflection tasks and self-assessment forms in the web-program |  |  |  |  |
| The support of the OT |  |  |  |  |
| The number of online meetings with the OT |  |  |  |  |
| The content of your activity plan |  |  |  |  |
| Follow-up support by the OT during the realization of the content of the activity plan |  |  |  |  |
| The number of meetings with the OT during the realization of your activity plan |  |  |  |  |
| Your own effort during SEE |  |  |  |  |
| The result you achieved from SEE |  |  |  |  |

Do you have any other comments on the content and design of SEE? If so, please describe them here:

Has SEE affected you negatively in any way or caused harm? If so, please describe how here:
