# Supplementary material for: A feasibility study of the internet-based intervention “Strategies for Empowering activities in Everyday life” (SEE 1.0) applied for people with stroke
Source: BMC Health Serv Res. 2025 Mar 4;25:330. doi: 10.1186/s12913-025-12456-8 (PMC11877923; doi:10.1186/s12913-025-12456-8)
Supplement: Supplementary file 3 — Supplementary Material 3. [file 12913_2025_12456_MOESM3_ESM.docx]

**The value of “Strategies for Empowering Activities in Everyday Life” (SEE)**

Please indicate to what extent the following statements about SEE agree with your experience by mark your answer with a cross in the appropriate box

|  |  |  |  |  |
| --- | --- | --- | --- | --- |
| **Statements about the value of SEE:** | **Strongly disagree** | **Agree to some extent** | **Agree** | **Strongly agree** |
| Have increased my knowledge of the importance of activities in everyday life to feel good and experience health |  |  |  |  |
| Has given me new insights into my pattern of activities in everyday life and my balance in activities and how they affect my well-being |  |  |  |  |
| Has helped me to "see" my activities in everyday life in a new way |  |  |  |  |
| Has given me insight into how I can change activities in my everyday life to achieve an active life under my new conditions |  |  |  |  |
| Has given me strategies that I can use/have used to change my everyday life |  |  |  |  |
| Has helped me take an active role in changing my life situation |  |  |  |  |
| Has given me an increased readiness to take on everyday challenges in the future |  |  |  |  |
| Has helped me in my change process in my new life situation |  |  |  |  |

Do you have any other comments on the values of SEE? If so, please describe them here:
