# Supplementary material for: A feasibility study of the internet-based intervention “Strategies for Empowering activities in Everyday life” (SEE 1.0) applied for people with stroke
Source: BMC Health Serv Res. 2025 Mar 4;25:330. doi: 10.1186/s12913-025-12456-8 (PMC11877923; doi:10.1186/s12913-025-12456-8)
Supplement: Supplementary file 4 — Supplementary Material 4. [file 12913_2025_12456_MOESM4_ESM.docx]

**Interview guide, group interview with occupational therapists who has delivered SEE**

*SEEs education program*

- Is there a need to develop the education program? If so, how?
- Does the education program provide enough support to prepare you for delivering SEE?

*SEEs intervention guide*

- Is there a need to develop the intervention guide? If so, how?
- Does the intervention guide provide enough support when delivering SEE?
- Has there been a need to deviate from the intervention guide? If so, how?

*SEEs intervention process*

- How has internet-based format worked?
- Is the number of modules suitable?
- Is there a need to develop the logical order of the modules? If so, how?
- Is the number of digital meetings with the clients suitable/ enough?
- Is there a need to develop the content of any part? If so, what and how?
- Is there something that needs to be changed, improved, added or done differently in the intervention process?

*General*

- Do you have any remining comments?
