# Supplementary material for: A feasibility study of the internet-based intervention “Strategies for Empowering activities in Everyday life” (SEE 1.0) applied for people with stroke
Source: BMC Health Serv Res. 2025 Mar 4;25:330. doi: 10.1186/s12913-025-12456-8 (PMC11877923; doi:10.1186/s12913-025-12456-8)
Supplement: Supplementary file 5 — Supplementary Material 5. [file 12913_2025_12456_MOESM5_ESM.docx]

**Interview guide, group interviews with clinic managers in organizations delivering SEE**

*SEE's feasibility, acceptability and value in the different organizations (you might have had different conditions in different organizations to deliver SEE)*

- What are your experiences with the feasibility of SEE? What benefits and challenges have you encountered?
- Based on the results, what are your reflections on the impact of SEE on clients and the occupational therapists work?
- Considering aspects such as procedures, resource consumption, management, and results,
- How does SEE function in terms of usability, accessibility, dose, adherence, and adaptation?
- Is there a need for further development in SEE? If so, what areas require improvement?

*The relevance and possibility of long-term implementation of SEE in your different organizations*

- In relation to your organization’s planned development and goals, is there a need to implement SEE? Why or why not?
- If so, what changes are needed for long-term implementation?
- What are the needs for education and educational resources?
- What are the needs for information, materials, and suitable channels for these?

*The potential of SEE in other contexts*

- Can SEE be relevant in other contexts? If so, where and for whom? What benefits and obstacles might exist in these contexts?
- How can barriers at different organizational levels and between different organizations be bridged to disseminate and transfer research results to other organizations?

*General*

- Is there anything else you would like to convey to us?
